# New data on the genus *Derops* Sharp (Coleoptera, Staphylinidae, Tachyporinae) from China with description of two new species

**DOI:** 10.3897/zookeys.317.5505

**Published:** 2013-07-19

**Authors:** Jie-Qiong Zhao, Li-Zhen Li

**Affiliations:** 1 Department of Biology, College of Life and Environmental Sciences, Shanghai Normal University, Shanghai, 200234, P. R. China

**Keywords:** Coleoptera, Staphylinidae, *Derops*, new species, key, China

## Abstract

Two new Chinese species of *Derops* are described: *Derops hainanus* **sp. n.** from Hainan and *Derops yunnanus* **sp. n.** from Yunnan. Females of *Derops punctipennis* Schülke and *Derops schillhammeri* Schülke are described for the first time and new provinces records of *Derops smetanai* Schülke and *Derops dingshanus* Watanabe are reported. The key to Chinese species of *Derops* published by Schülke 2003 is modified to include the new species.

## Introduction

The genus *Derops* Sharp, 1889, is a small genus with 17 species in the world. Most of them are known from east and south-east Asia (Russian Far East, Korea, Japan, China, Vietnam, India) and a single species occurs in the eastern United States. Presently, ten species have been recorded in China, named *Derops longicornis* Sharp, 1889, *Derops coreanus* Watababe, 1969, *Derops lisae* Smetana, 1995, *Derops dingshanus* Watanabe, 1999, *Derops vietmanicus* Watanabe, 1996, *Derops nitidipennis* Schülke, 2000, *Derops smetanai* Schülke, 2003, *Derops schillhammeri* Schülke, 2003, *Derops rougemonti* Schülke, 2003, and *Derops punctipennis* Schülke, 2003. During our ongoing study on the genus *Derops*, two new species are recognized, the females of *Derops punctipennis* Schülke and *Derops schillhammeri* Schülke are discovered for the first time, and Zhejiang and Chongqing are new provinces records for *Derops smetanai* Schülke and *Derops dingshanus* Watanabe respectively.

## Material and methods

All measurements are given in millimeters. The following abbreviations are used in the text:

BL – The length of the body from front margin of head to the apex of the abdomen; FL – The length of the body from front margin of head to elytra end; HL – The length of the head from the clypeal anterior margin to the head base; HW – The maximum width of the head with eyes; PL – The length of the pronotum along the midline; PW – The maximum width of the pronotum; EL – The length of the elytra from the apex of the scutellum to the elytral posterior margin; EW – The maximum width of the elytra; SL – The length of elytra suture; ED – The diameter of eyes in longitudinal direction from lateral; TL – The longitudinal length of temple.

The specimens were collected from the leaf litter along the mountain stream by sifting. For examination of the male genital organ, the abdominal segments were detached from the body after softening in hot water. The aedeagus and other dissected parts were mounted in Euparal (Chroma Gesellschaft Schmidt, Koengen, Germany) on plastic slides. Photos of sexual characters were taken by a Canon G9 camera attached to an Olympus SZX 16 stereoscope.

The types are deposited in the Insect Collection of Shanghai Normal University, Shanghai, China (SNUC).

## Descriptions

### Derops hainanus

Zhao & Li sp. n.

urn:lsid:zoobank.org:act:15DFD8A0-9861-4B0B-9F7E-90DA74BAEEBA

http://species-id.net/wiki/Derops_hainanus

Figs 1A , 2

#### Type locality.

Hainan Prov., China

#### Type material.

(1 ♂). HOLOTYPE: ♂, labeled ‘China: Hainan Prov. / Changjiang County / Bawangling Nature Reserve / 11.iv.2010, alt. 1,000 m / Zi-Wei Yin leg.’.

#### Description.

Measurements and ratios. BL: 4.23; FL: 2.89; HL: 0.46; HW: 0.78; PL: 0.85; PW: 0.93; EL: 1.59; EW: 1.13; SL: 1.31; ED: 0.37; TL: 0.17; HW/HL: 1.70; PW/PL: 1.09; EL/EW: 1.41; PW/HW: 1.19; EL/PL: 1.87; EW/PW: 1.22; ED/TL: 2.18.

This species is assigned to *Derops longicornis* group based on the following characteristics: elytra finely and densely punctured, not or little shiny; male sternite VII deep emarginated on the posterior margin, two additional granules fields next to the posterior margin with short, strong and blunt seta.

**Figure 1.**
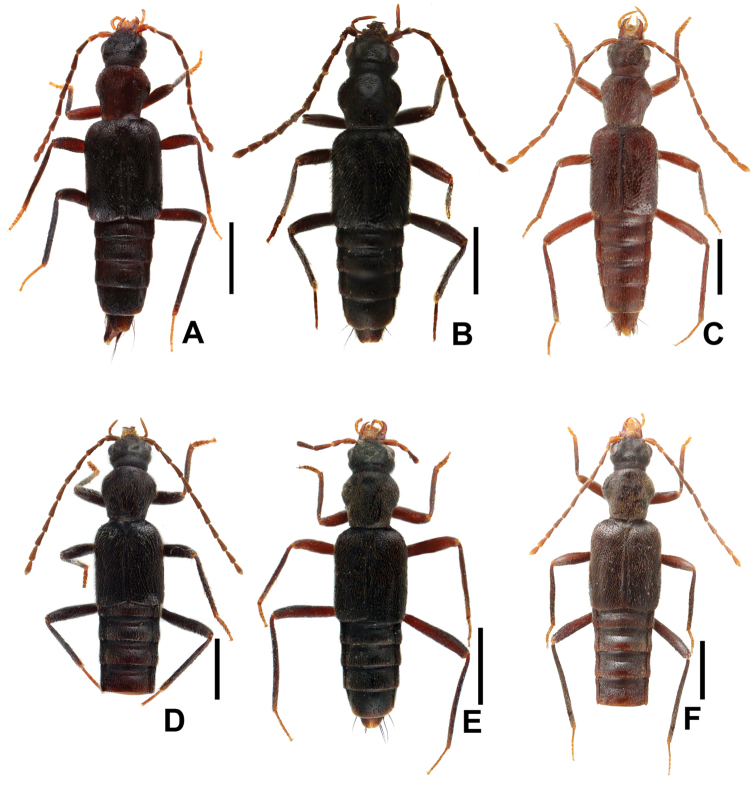
Habitus of *Derops* spp., **A** *Derops hainanus* Zhao & Li, sp. n. **B** *Derops yunnanus* Zhao & Li, sp. n. **C** *Derops punctipennis* Schülke **D** *Derops schillhammeri* Schülke **E** *Derops smetanai* Schülke; **F** *Derops dingshanus* Watanabe. Scale bars: 1.0 mm.

Body (Fig. 1A). Uniformly reddish black to reddish brown and moderately shining; mouthparts including maxillary and labial palpi, apical two and first two antennal segments, tarsi yellowish brown; pronotum, the rest of antennae and legs except for tarsi reddish brown; elytra light to dark brunneous. Body narrowly elongate, subparallel-sided and somewhat convex; sides of abdomen gradually narrowed from base to apex.

Head distinctly transverse and impressed, broader across eyes than long (HW/HL: 1.70), with shining punctures moderately coarse and dense, without microsculpture, and surface covered with short, sparse, fine yellowish brown pubescence, but almost deprived of pubescence close to neck. Eyes relatively large (ED/TL: 2.18) and slightly prominent laterad, postocular region gently arcuate and loosely contracted at neck. Antenna filiform and almost extending to the middle of elytra, all the segments with pubescence slightly dilated apicad, 1st segment robust, 2nd the shortest and as twice as broad, 3rd to 6th equal in both broad and distinctly longer than broad, 7th to 8th equal in both length and width, 9th to 10th equal in width, and 9th longer than 10th, 11th distinctly longer and narrower than 10th, excavated at the apex.

Pronotum obcordate and convex, slightly transverse (PW/PL: 1.09), distinctly broader than head (PW/HW: 1.19); expanded laterally in anterior one fifth, arcuate in anterior two-fifths and almost straight in posterior three-fifths, anterior angles bluntly angulate and invisible from above; posterior ones rectangular. surface on both sides with punctate as head, most interspaces between punctures somewhat less than diameters of punctures, covered with sparse, fine, moderately long, yellowish brown pubescence all over, without sculpture; providing with a shallow depression at the middle.

Elytra oblong, visibly longer than broad (EL/EW: 1.41), obviously longer (EL/PL: 1.87) and somewhat broader (EW/PW: 1.22) than pronotum; lateral sides nearly parallel, posterior margin emarginate at the middle, posterior angles broadly rounded; surface densely, fine punctate, more sparser than head and elytra, transverse distances between punctures mostly twice than diameters of punctures, and shorter pubescence than pronotum, without sculpture, possessing two shallow and longitudinal depressions, along suture and lateral side respectively; epipleura each bearing a fine longitudinal keel, which is abbreviated behind shoulder. Scutellum small and ligulate.

Abdomen subcylindrical, gradually tapering towards apex; 4th to 7th tergite each transversely depressed along the base, punctation relatively coarse before the depression; each superficially with densely short fine pubescence.

**Figure 2.**
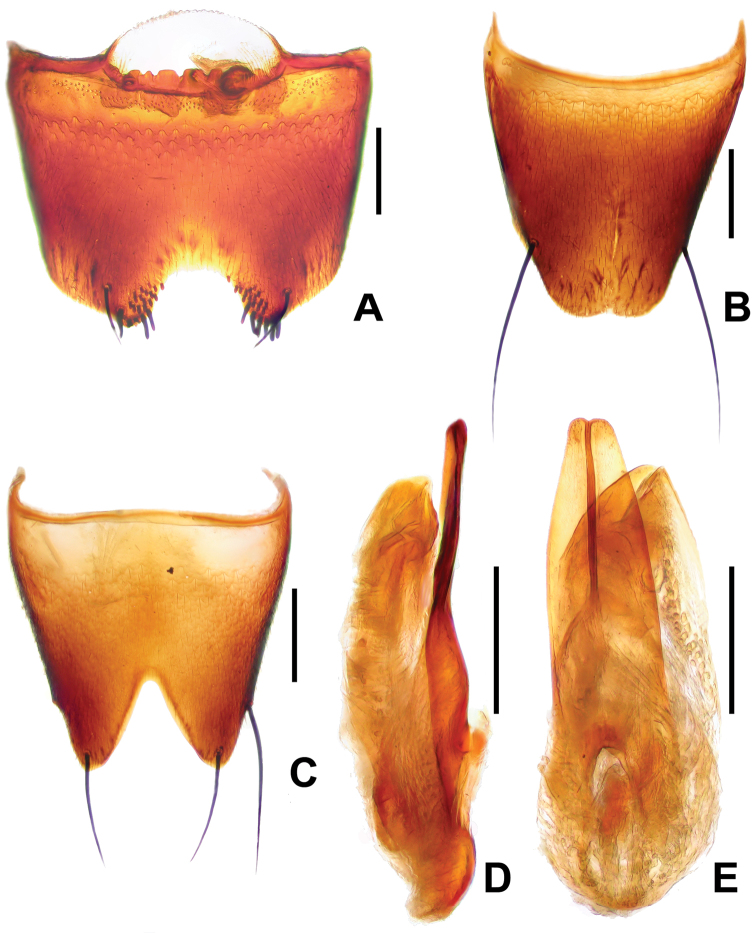
*Derops hainanus* Zhao & Li, sp. n. **A** male sternite VII **B** male tergite VIII **C** male sternite VIII **D** aedeagus in lateral view **E** aedeagus in ventral view. Scale bars: **A–C** and **F–G**: 0.2 mm; **D–E**: 0.25 mm.

Male. Sternite VII (Fig. 2A) deeply, wide, almost “U” shaped medio-apical emargination, depth approximately two-sevenths of the length, and armed with six short blackish seta and a long, black seta on each side of the emargination; with field of about 15 granules on each side of emargination. Tergite VIII (Fig. 2B) with shallow medio-apical emargination and with two long, strong, black setae at each lateral margin apical third. Sternite VIII (Fig. 2C) wide and very deep, blunt triangular emargination at the middle of posterior margin, depth about two-fifths of the length, surface with two long, strong, black seta at each lateral margin. Genital organ (Fig. 2D, E) long oval, slightly sclerotized; with median lobe no longer than lateral lobes combined; viewed dorsally, lateral lobes elongate, symmetrical, evenly narrowed to obtuse apices; viewed laterally, lateral lobes hardly bent ventrally, apical portion slightly barbed.

Female. Unknown.

#### Distribution.

China: Hainan Prov.

#### Etymology.

The name of the new species is derived from that of the type locality.

#### Remarks.

The new species may be readily distinguished from the rest species of *Derops longicornis* group by the following characteristics: male sternite VII field of not exceeding the half of medio-apical emargination depth, while in other species exceeding the half of medio-apical emargination depth, and even extended to emargination apex.

### Derops yunnanus

Zhao & Li sp. n.

urn:lsid:zoobank.org:act:E67A0382-5FDF-444E-B866-16D56D606E93

http://species-id.net/wiki/Derops_yunnanus

Figs 1B , 3

#### Type locality.

Yunnan Prov., China

#### Type material.

(6 ♂♂, 2 ♀♀). HOLOTYPE: ♂, labelled ‘China: Yunnan Prov. / Xianggelila County / Hutiaoxia Nature Reserve / Jinxing Village / 22.iv.2005, alt. 2,300 m / Hao Huang leg.’; PARATYPES: 4 ♂♂, 2 ♀♀, same label data as holotype; 1 ♂, same, but ‘22.iv.2005.’.

#### Description.

Measurements and ratios. BL: 4.34–4.50; FL: 2.84–3.11; HL: 0.45–0.46; HW: 0.72–0.83; PL: 0.85–0.89; PW: 0.93–0.95; EL: 1.39–1.50; EW: 1.22–1.33; SL: 1.06–1.22; ED: 0.28–0.31; TL: 0.11–0.15; HW/HL: 1.60–1.84; PW/PL: 1.07–1.09; EL/EW: 1.13–1.17; PW/HW: 1.14–1.32; EL/PL: 1.56–1.69; EW/PW: 1.28–1.40; ED/TL: 2.07–2.55.

This species is assigned to *Derops nitidipennis* group based on its distinctive rough and extensive puncturing; male sternite VII is emarginate just flat at the posterior margin, the additional fields consist of long, apically pointed peg-like setae.

Body (Fig. 1B). Uniformly piceous-black to black and moderately shining; mouthparts including maxillary and labial palpi, antennal segments and tarsi reddish brown. Body narrowly elongate, subparallel-sided and somewhat convex; sides of abdomen gradually narrowed from base to apex.

Head distinctly transverse and impressed, broader across eyes than long (HW/HL: 1.60–1.84), with shining punctures moderately coarse and dense, without microsculpture, and surface covered with long brown pubescence and almost glossy near neck. Eyes relatively large (ED/TL: 2.07–2.55) and prominent laterad, postocular region gently arcuate and loosely contracted at neck. Antenna filiform and exceeding the middle of elytra, all the segments with pubescence slightly dilated apicad, 1st segment longest, 2nd the shortest and as twice as broad, 1st to 4th equal in both broad and distinctly longer than broad, 5th to 9th equal in both length and width and length more than three times the width, 10th to 11th equal in both length and width and length at most twice the width, 11th excavated at the apex.

Pronotum obcordate and convex, slightly transverse (PW/PL: 1.07–1.09), distinctly broader than head (PW/HW: 1.14–1.32); expanded laterally widest in anterior one-third, arcuate in anterior two-thirds and almost straight in posterior third, anterior angles bluntly angulate and invisible from above; posterior ones almost rectangular. surface on both sides with shining moderately coarse and sparser punctate than those of head, most interspaces between punctures somewhat less than half of diameters of punctures, covered with sparse, fine, yellowish brown pubescence all over, without sculpture; providing with a shallow depression at the middle of anterior margin.

Elytra oblong, slightly longer than broad (EL/EW: 1.13–1.17), obviously longer (EL/PL: 1.56–1.69) and somewhat broader (EW/PW: 1.28–1.40) than pronotum; lateral sides nearly parallel, posterior margin emarginate at the middle, posterior angles broadly rounded; surface densely, coarse punctate, more sparser, but shallower than head and elytra, transverse distances between punctures mostly one to 2.5 times than diameters of punctures, and longer pubescence than pronotum, without sculpture, possessing two shallow and longitudinal depressions, one on each side of suture and the other on each lateral side; epipleura each bearing a fine longitudinal keel, which is abbreviated behind shoulder. Scutellum small and ligulate, and with dense, yellowish brown pubescence.

Abdomen subcylindrical, gradually tapering towards apex; 4th to 7th tergite each transversely depressed along the base, surface uneven depression, and providing with moderately coarse and dense punctation before depression, fine and shining punctation after depression; each superficially with dense, short and fine pubescence.

**Figure 3.**
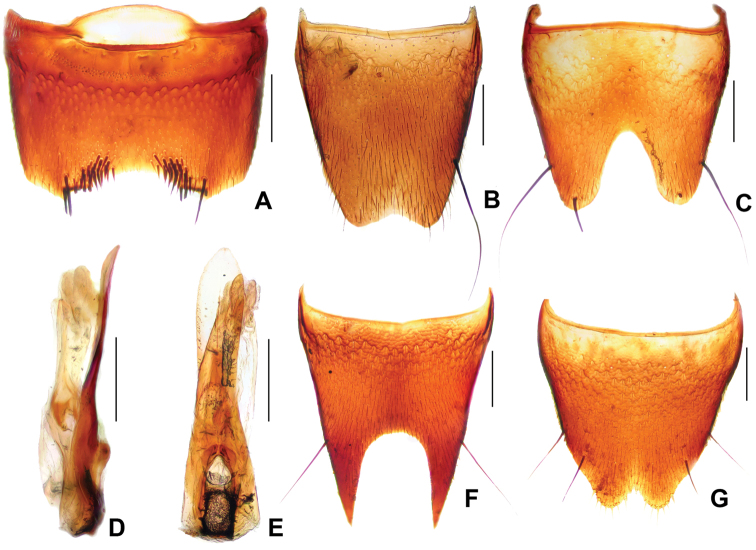
*Derops yunnanus* Zhao & Li, sp. n. **A** male sternite VII **B** male tergite VIII **C** male sternite VIII **D** aedeagus in lateral view **E** aedeagus in ventral view **F** female tergite VIII **G** female sternite VIII. Scale bars: **A–C** and **F–G**: 0.2 mm; **D–E**: 0.25 mm.

Male. Sternite VII (Fig. 3A) shallow, wide medio-apical emargination, and armed with six short rigid blackish cilia and a long, black seta on each side of the emargination; with field of about 20 apically acute peg-like setae on each side of emargination. Tergite VIII (Fig. 3B) with shallow medio-apical emargination and with two long, strong, black setae at each lateral margin two-thirds. Sternite VIII (Fig. 3C) wide and very deep, blunt triangular emargination at the middle of posterior margin, emargination almost extending to the middle, surface with two long, strong, black seta at each lateral margin. Genital organ (Fig. 3D, E) long oval, slightly sclerotized, median lobe shorter than lateral lobes combined; lateral lobes elongate, symmetrical, expand in one half and narrowed to obtuse apices; viewed laterally, lateral lobes hardly bent ventrally, apical portion slender.

Female. Tergite VIII (Fig. 3F) deep, broadly, “U” shaped excised at the middle of posterior margin, one very long black seta at each lateral margin at about apical four-sevenths, almost extending the middle of tergite. Sternite VIII (Fig. 3G) broadly emarginate at the middle of posterior margin and fringed with seven yellowish pubescence at the latero-posterior parts, and respectively with two long, strong, black setae at each lateral margin in apical three-fifths and four-fifths.

#### Distribution.

China: Yunnan Prov.

#### Etymology.

The specific name is derived from “Yunnan”, the province of the type locality.

#### Remarks.

The new species is similar to *Derops shuckburghae* Rougemont described from Thailand, but it may be readily distinguished from the latter one by the following characteristics: head larger and relatively broader with HW/HL: 1.60–1.84 (1.54 in *Derops shuckburghae*); elytra shorter than pronotum with EL/PL: 1.56–1.69 (1.72 in *Derops shuckburghae*), and punctation larger and denser, transverse distances between punctures mostly one to 2.5 times than diameters of punctures (transverse distances 2 to 3 times greater than diameters of punctures in *Derops shuckburghae*); male sternite VII with field of about 20 apically acute peg-like setae on each side of medio-apical emargination (only 6–7 peg-like setae in *Derops shuckburghae*); aedeagus lateral lobes symmetrical, expand in one half and narrowed to obtuse apices (slightly asymmetrical, evenly narrowed to obtuse apices in *Derops shuckburghae*); female tergite VIII excised broader in anterior, almost extending the middle of tergite (excised narrower in anterior, clearly not extending the middle of tergite in *Derops shuckburghae*).

### Derops punctipennis

Schülke, 2003

http://species-id.net/wiki/Derops_punctipennis

Figs 1C , 4

Derops punctipennis Schülke 2003: 471.

#### Specimens examined.

(4 ♂♂, 5 ♀♀). 2 ♂♂, 2 ♀♀, labelled ‘China: Zhejiang Prov. / Anji County / Mt. Longwangshan / 24.iv.2006, alt. 300–500 m / Liang Tang leg.’; 1 ♂, 1 ♀, labelled ‘China: Zhejiang Prov. / Linan City / Qingliangfeng Nature Reserve / 10.v.2005, alt. 1,080 m / Li-Long Zhu & Jin-Wen Li leg.’; 1 ♀, same, but ‘9.v.2005, alt. 1,050–1,070 m’; 1 ♂, 1 ♀, labeled ‘China: Zhejiang Prov. / Xianju County / Danzhu Village / 2.vi.2006, alt. 450–600 m / Jin-Wen Li & Shan-Jia Shen leg.’.

#### Description.

Measurements. BL: 4.56–6.06; FL: 2.98–3.39.

**Figure 4.**
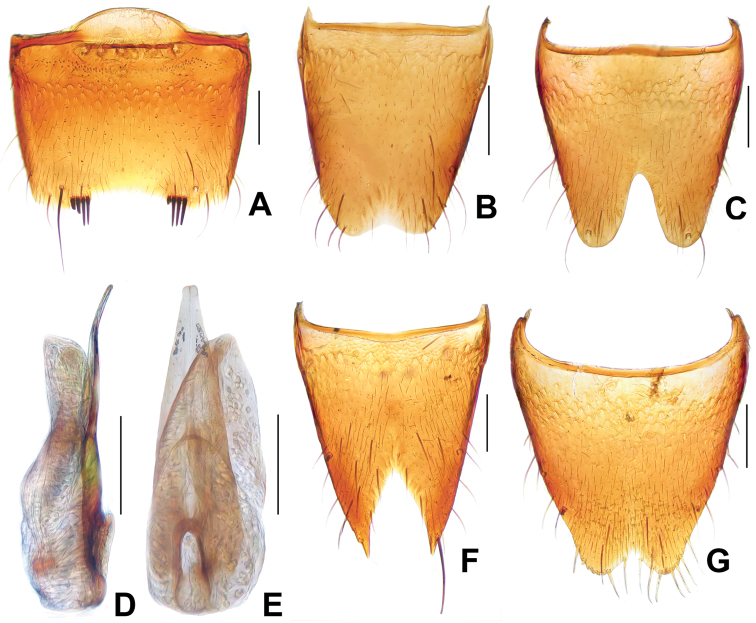
*Derops punctipennis* Schülke. **A** male sternite VII **B** male tergite VIII **C** male sternite VIII **D** aedeagus in lateral view **E** aedeagus in ventral view **F** female tergite VIII **G** female sternite VIII. Scale bars: **A–C** and **F–G**: 0.2 mm; **D–E**: 0.25 mm.

Female. Tergite VIII (Fig. 4F) with wide and very deep, acute triangular medio-apical emargination, one very long, strong, black seta at apical margin at each side of medio-apical ernargination. Sternite VIII (Fig. 4G) broadly emarginate eat the middle of posterior margin and bearing six or seven yellowish cilia at the latero-posterior parts.

#### Distribution.

China: Fujian, Zhejiang.

#### Remarks.

The species was previously described based on male specimens from Fujian Prov., and this is the first record of its female. It is very similar to *Derops smetanai* Schülke, from Fujian (Schülke 2003: 467, figs 1–9). Males may be readily distinguished by the shape of sternite VII emargination and by evenly narrowed, slightly bend to obtuse apices of aedeagus lateral lobes. Females may be easily distinguished by the shape of tergite VIII emargination.

### Derops schillhammeri

Schülke, 2003

http://species-id.net/wiki/Derops_schillhammeri

Figs 1D , 5

Derops schillhammeri Schülke 2003: 470.

#### Specimens examined.

(6 ♂♂, 3 ♀♀). 3 ♂♂, 1 ♀, labeled ‘China: Hubei Prov. / Wufeng County, / Houhe Nature Reserve / 29.iv.2004, alt. 1,000 m / Li-Zhen Li leg.’; 1 ♀, same, but ‘30.iv.2004.’; 3 ♂♂, 1 ♀, labelled ‘China: Zhejiang Prov. / Xianju County / Danzhu Village / 2.vi.2006, alt. 450–600 m / Jin-Wen Li & Shan-Jia Shen leg.’.

#### Description.

Measurements. BL: 4.95–5.56; FL: 2.77–3.28.

**Figure 5.**
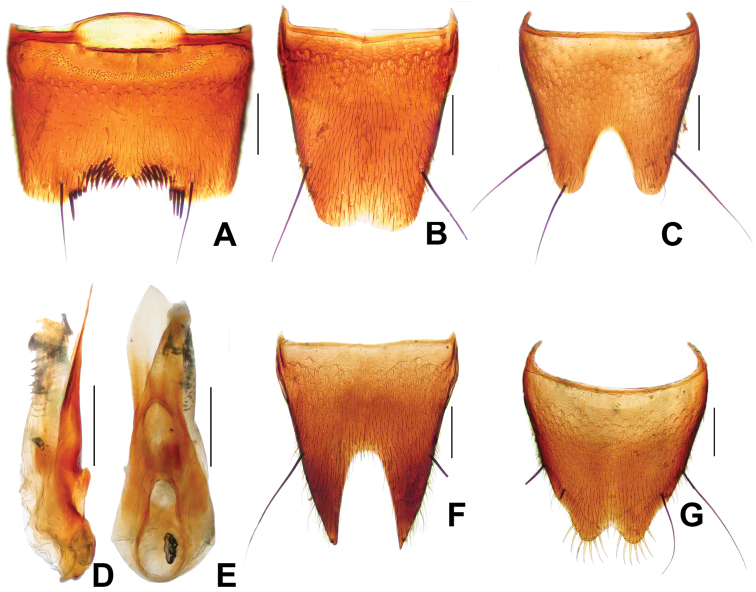
*Derops schillhammeri* Schülke. **A** male sternite VII **B** male tergite VIII **C** male sternite VIII **D** aedeagus in lateral view **E** aedeagus in ventral view **F** female tergite VIII; **G** female sternite VIII. Scale bars: **A–C** and **F–G**: 0.2 mm; **D–E**: 0.25 mm.

Female. Tergite VIII (Fig. 5F) with wide and very deep, almost “U” shaped medio-apical emargination, one very long, strong, black seta at the middle of each lateral margin of emargination. Sternite VIII (Fig. 5G) broadly emarginate at the middle of posterior margin and provided with seven or eight yellowish cilia at the latero-posterior parts, and with two long, strong, black setae at each lateral margin in apical third.

#### Distribution.

China: Hubei, Jiangxi, Zhejiang.

#### Remarks.

**Figure 6.**
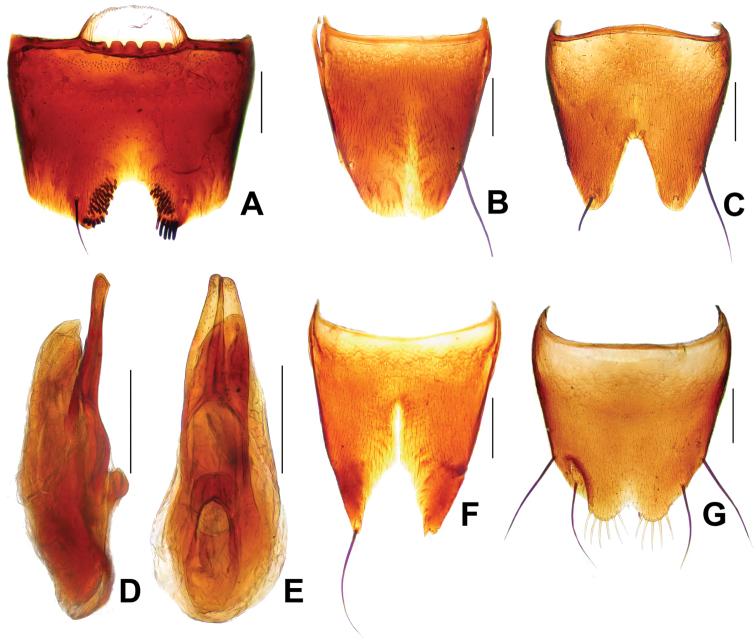
*Derops smetanai* Schülke. **A** male sternite VII **B** male tergite VIII **C** male sternite VIII **D** aedeagus in lateral view **E** aedeagus in ventral view **F** female tergite VIII; **G** female sternite VIII. Scale bars: **A–C** and **F–G**: 0.2 mm; **D–E**: 0.25 mm.

The species was previously described based on male specimens from Jiangxi Prov., and this is the first record of its female. The male of *Derops schillhammeri* Schülke previously known from Jiangxi Prov., China. It is very similar to *Derops shuckburghae* Rougemont, from Thailand (Rougemont 2003: 998-999, plate 1, 2 (figs 1, 3-6, 8)). Females may be easily distinguished by the depth of tergite VIII emargination almost half of itself and not very broad apices.

### Derops smetanai

Schülke, 2003

http://species-id.net/wiki/Derops_smetanai

Figs 1E , 6

Derops smetanai Schülke 2003: 467.

#### Specimens examined.

(15 ♂♂, 14 ♀♀). 10 ♂♂, 11 ♀♀, labeled ‘China: Zhejiang Prov. / Xianju County / Danzhu Village / 2.vi.2006, alt. 450–600 m / Jin-Wen Li & Shan-Jia Shen leg.’; 1 ♂, labeled ‘China: Zhejiang Prov. / Panan County / Dapanshan Nature Reserve / 7.vi.2006, alt. 550–700 m / Jin-Wen Li & Shan-Jia Shen leg.’; 1 ♂, 1 ♀, labelled ‘China: Zhejiang Prov. / Xianju County / Shangjing Village / 3.vi.2006, alt. 450–650 m / Jin-Wen Li & Shan-Jia Shen leg. ’; 1 ♂, labeled ‘China: Zhejiang Prov. / Taishun County / Wuyanling Nature Reserve / 10.v.2004, alt. 700–850 m / Jia-Yao Hu, Liang Tang & Li-Long Zhu leg. ’; 1 ♀, labeled ‘China: Zhejiang Prov. / Qingyuan County / Baishanzu Nature Reserve / 4.v.2004, alt. 1,050 m / Jia-Yao Hu, Liang Tang & Li-Long Zhu leg.’; 1 ♂, 1 ♀, same, but ‘5.v.2004, alt. 1,200-1,360 m.’; 1 ♂, labeled ‘China: Zhejiang Prov. / Kaihua County / Mt. Gutianshan Nature Reserve / 5–7.v.2005, alt. 800–900 m / Li-Long Zhu & Jin-Wen Li leg.’.

#### Measurements.

BL: 4.23–5.00; FL: 2.78–3.17.

#### Distribution.

China: Jiangxi, Zhejiang.

#### Remarks.

The species is firstly recorded in Zhejiang.

### Derops dingshanus

Watanabe, 1999

http://species-id.net/wiki/Derops_dingshanus

Figs 1F , 7

Derops dingshanus Watanabe 1999: 253; Schülke 2000: 915; Zheng 2002: 38.
Derops puetzi Schülke 1999: 345; Schülke 2003: 466.

#### Specimens examined.

(16 ♂♂, 21 ♀♀). 3 ♂♂, 2 ♀♀, labeled ‘China: Zhejiang Prov. / Panan County / Dapanshan Nature Reserve / 7.vi.2006, alt. 550–700 m / Jin-Wen Li & Shan-Jia Shen leg.’; 1 ♂, 1 ♀ labeled ‘China: Zhejiang Prov. / Xianju County / Shangjing Village / 3.vi.2006, alt. 450–650 m / Jin-Wen Li & Shan-Jia Shen leg. ’; 2 ♂♂, 4 ♀♀ labeled ‘China: Zhejiang Prov. / Linan County / Mt.Tianmu / 11–15.vi.2006, alt. 300–400 m / Jia-Yao Hu & Liang Tang leg. ’; 1 ♂, 1 ♀, labeled ‘China: Zhejiang Prov. / Linan County / East Mt.Tianmu / 13.iv.2011, alt. 1,050–1,150 m / Zhong Peng & Jian-qing Zhu leg. ’; 6 ♂♂, 6 ♀♀, labeled ‘China: Jiangsu Prov. / Nanjing City / Mt.Zijin / 14.v.2006, alt. 400 m / Liang Tang leg. ’; 1 ♂, labeled ‘China: Zhejiang Prov. / Zhuji City / Wuxie / 19.iii.2005, alt. 300–400 m / Liang Tang leg. ’; 1 ♂, labeled ‘China: Jiangxi Prov. / Mt. Sanqingshan National Park / 4.v.2005, alt. 700–1,000 m / Jia-Yao Hu & Liang Tang leg. ’; 1 ♂, labeled ‘China: Chongqing City. / Chengkou County / Mt. Dabashan / 4.iv.2008, alt. 1,830 m / Hao Huang & Wang Xu leg.’; 2 ♀♀, labeled ‘China: Zhejiang Prov. / Anji County / Mt. Longwangshan / 24.iv.2006, alt. 300–500 m / Liang Tang leg.’; 1 ♀, labeled ‘China: Zhejiang Prov. / Wenzhou City / Mt. Yandangshan / 9.v.2006, alt. 50–150 m / Jin-Wen Li & Shan-Jia Shen leg.’; 1 ♀, labeled ‘China: Zhejiang Prov. / Ningbo City / Mt. Xishan / 3.iv.2013, alt. 200 m / Jian-Qing Zhu leg.’; 1 ♀, labeled ‘China: Zhejiang Prov. / Kaihua County / Mt. Gutianshan Nature Reserve / 5–7.v.2005, alt. 800–900 m / Li-Long Zhu & Jin-Wen Li leg.’; 1 ♀, labeled ‘China: Zhejiang Prov. / Zhuji City / Caota Town Nature Reserve / 20.xi.2011, alt. 100 m / Tie-Xiong Zhao leg.’; 1 ♀, labeled ‘China: Guizhou Prov. / Suiyang County / Kuankuoshui Nature Reserve / 11.viii.2010, alt. 1,400 m / Zi-Wei Yin & Ting Feng leg.’.

#### Measurements.

BL: 4.50–5.06; FL: 2.90–3.22.

**Figure 7.**
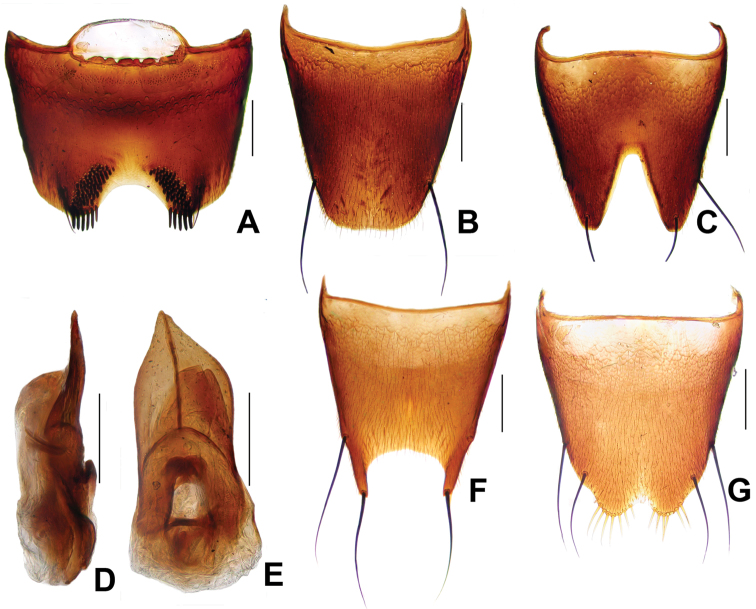
*Derops dingshanus* Watanabe. **A** male sternite VII **B** male tergite VIII **C** male sternite VIII **D** aedeagus in lateral view **E** aedeagus in ventral view **F** female tergite VIII **G** female sternite VIII. Scale bars: **A–C** and **F–G**: 0.2 mm; **D–E**: 0.25 mm.

#### Distribution.

China: Chongqing, Guizhou, Jiangsu, Shaanxi, Sichuan, Zhejiang.

#### Remarks.

The species is firstly recorded in Chongqing.

### The recently published key of Chinese *Derops* (Schülke, 2003) should be modified as following to accommodate the new species:

**Table:** 

5	Eyes larger, more than twice as long as the temples. Aedeagus with slender, apically slightly tapered parameres	5a
–	Eyes small, less than twice as long as the temples. Aedeagus with very slender or broad, apically pointed parameres. Female tergite VIII much broader and deeper excised, side parts very narrow and apically pointed	6
5a	Elytra punctures larger and denser, transverse distances between punctures mostly one to 2.5 times than diameters of punctures. Male sternite VII with field of about 20 apically acute peg-like setae on each side of emargination. Aedeagus lateral lobes symmetrical, expand in one half and narrowed to obtuse apices. China: Yunnan	*Derops yunnanus* sp. n.
–	Elytra punctures smaller and sparser, transverse distances 2 to 3 times greater than diameters of punctures. Male sternite VII with field of only 6–7 peg-like setae on each side of emargination. Aedeagus lateral lobes slightly asymmetrical, evenly narrowed to obtuse apices. Thailand	*Derops shuckburghae* Rougemont
7	Very large and robust species, pronotum broader (PW> 1.00 mm). Male sternite VII with posterior margin bristles, without elongate field (fig. 14). Aedeagus (fig. 16) large (> 1 mm) with long parallel parameres. China: Fujian	*Derops rougemonti* Schülke
–	Slightly smaller species, pronotum narrower (PW <1.00 mm). Male sternite VII with elongate field. Aedeagus small (<1.00 mm)	7a
7a	Male sternite VII (fig. 2B) granules field not exceeding the half of medio-apical emargination depth. Female unknown. China: Hainan	*Derops hainanus* sp. n.
–	Male sternite VII granules field exceeding the half of medio-apical emargination depth	8

## Supplementary Material

XML Treatment for Derops hainanus

XML Treatment for Derops yunnanus

XML Treatment for Derops punctipennis

XML Treatment for Derops schillhammeri

XML Treatment for Derops smetanai

XML Treatment for Derops dingshanus
